# Selection of the Activated Carbon Type for the Treatment of Landfill Leachate by Fenton-Adsorption Process

**DOI:** 10.3390/molecules25133023

**Published:** 2020-07-02

**Authors:** Liliana San-Pedro, Roger Méndez-Novelo, Emanuel Hernández-Núñez, Manuel Flota-Bañuelos, Jorge Medina, Germán Giacomán-Vallejos

**Affiliations:** 1 Engineering Faculty, Autonomous University of Yucatan, P.O. Box 150, Mérida, Mexico; mnovelo@correo.uady.mx (R.M.-N.); manuel.flota@correo.uady.mx (M.F.-B.); jorge.medina@correo.uady.mx (J.M.); giacoman@correo.uady.mx (G.G.-V.); 2 Center of Research and Advanced Studies of the National Polytechnic Institute, P.O. Box 73, Merida Unit, Mexico; emanuel.hernandez@cinvestav.mx

**Keywords:** adsorption, activated carbon, isotherm, leachate, Fenton

## Abstract

Sanitary landfill leachates usually have characteristics that depend on the region where they are generated and according to the age of the landfill, which is why a unique treatment for their sanitation has not been found. However, the adsorption preceded by the Fenton process has been proven to be highly efficient at removing contaminants. In this study, the adsorptive capacity of two types of activated carbon, granular and powdered, was analyzed to determine which was more efficient in the adsorption stage in the Fenton-adsorption process. Likewise, its behavior was analyzed using three isotherm models (Langmuir, Freundlich and Temkin), testing the raw leachate and the Fenton-treated one with both carbons. The adsorption that is carried out on the carbons is better adjusted to the Freundlich and Temkin models. It concludes that multilayers, through the physical adsorption, carry out the adsorption of pollutants on the surface of the carbons. The results show that, statistically, granular activated carbon is more efficient at removing chemical oxygen demand (COD), and powdered activated carbon removes color better. Finally, an adsorption column was designed for the Fenton-adsorption process that was able to remove 21.68 kgCOD/kg carbon. Removal efficiencies for color and COD were >99%.

## 1. Introduction

The nature of landfill leachate depends on a wide variety of factors. Although each leachate has its own characteristics, they can be classified as young, medium or mature, regarding the age of the landfill. A particular and unique treatment that is effective in treating the many variants of leachates has not been found; however, the Fenton-adsorption process has been found to be highly efficient in leachates with mixed characteristics [1]. Adsorption is used as a stage of an integrated physical-chemical-biological process to treat leachate from landfills or simultaneously with a biological process [2,3]. Theoretically, the increase in temperature decreases the degree of adsorption and vice versa. The adsorption of solutes involves establishing a balance between the amount adsorbed on the surface and the concentration of substance in the solution [4]. Some adsorbents are more specific than others in attracting substances to the surface; organic pollutants are efficiently removed by activated carbon [5]; therefore, the most frequently used adsorbent is granular or powdered-activated carbon. Carbon adsorption allows to remove 50–70% chemical oxygen demand (COD) and ammoniacal nitrogen [2].

Activated carbon has a microporous and homogeneous structure, which gives it a large surface area [6,7]. Various raw materials for activated carbon have been tested to be efficient for the removal of contaminants, as well as economically viable—for example: sawdust, sugarcane bagasse, rice husk ash, eucalyptus bark, among others [8]. In the study of Xing et al. 2008 [9], four different types of activated carbon were tested: wood-based granular activated carbon (GAC), carbon-based GAC, wood-based powdered activated carbon (PAC) and carbon-based PAC to treat synthetic landfill leachate. The results showed that the organic matter removal efficiency is corresponding to the surface area of the activated carbon. The carbon-based PAC has a larger surface area to adsorb organic pollutants from the synthetic leachate; therefore, it performs better removal efficiency than the other activated carbons.

Currently, other adsorbent materials have been synthesized and applied mainly for the removal of heavy metal and specific substances such as acetaminophen or bisphenol-A—for example, graphene oxides [10,11,12], carbon nanotubes [13,14,15] or adsorbent composites [16,17]. Furthermore, magnetized materials such as magnetic graphene oxide or iron oxide magnetic nanoparticle have been reported with good removal efficiency for humic acids in general (98% and 96%, respectively), among other contaminants [18]. These materials could be a viable option for landfill treatment.

Adsorption is the concentration of a solute on the surface of a solid; this phenomenon occurs when the surface is in contact with a solution. The substance that is concentrated on the surface (or is adsorbed) is called “adsorbate”, and the adsorbent phase is called “adsorbent”.

It is currently recognized that there are two main types of adsorption: physical adsorption (physisorption), where adsorption is relatively weak, caused by the forces of Van Der Waals, and chemical adsorption (chemisorption), in which chemical bonds (mainly covalent) occur, making the adsorbate difficult to remove [19,20]. Physisorption plays a minor role in catalysis, except for some special types of reactions involving free or radical atoms, and may also be reversible, favoring the reuse of the adsorbent. In chemisorption, adsorbed molecules are attached to the surface by covalent forces of the same type as those occurring between the atoms of the molecules. Both types of adsorption can occur in some processes. Various surface investigations of known area have confirmed that chemisorption ceases after the unimolecular layer is formed, but physisorption can give rise to additional layers. 

In the adsorption process, equilibrium is reached when the sorption and desorption rates are equalized, at which point, the adsorption capacity of the adsorbent is exhausted. The theoretical adsorption capacity of a pollutant by means of an adsorbent can be established by calculating the adsorption isotherm [21], where the amount of adsorbed matter is determined as a function of the concentration of the adsorbate (C) at constant temperature (T).

**Figure 1:**
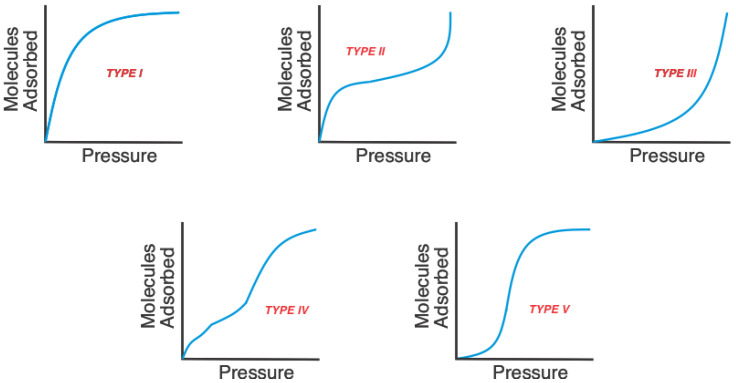
The five types of adsorption isotherms in the classification of Brunauer, Emett and Teller. Adapted from Brenner, 2013 [13].

The International Union of Pure and Applied Chemistry (IUPAC) recognizes six types of isotherms; however, according to the classification of Brunauer, Emett and Teller [22], adsorption processes can be classified into five (Figure 1). In chemisorption cases, only type I isotherms are present, while in physisorption, all five cases occur. 

The Freundlich; Langmuir; Brunauer, Emmett and Teller (BET) and Temkin models, among others, are used to describe the experimental isotherms. The information provided by the models is about the surface properties, the adsorption mechanism and the interaction between the adsorbent and the adsorbate [21,23].

The Langmuir isotherm analyzes the formation of the monolayer on the adsorbent based on the fact that the adsorption sites are finite and that, once they are filled, it will no longer be possible to adsorb more molecules [6,24]. Equation (1) represents the Langmuir adsorption model. The constants a and b are characteristics of the system under consideration and are evaluated from the experimental data.

(1) $$\frac{X}{M}=\frac{a*b*C_{e}}{1+bC_{e}}$$

In linear form, it can be expressed as:

(2) [1XM]=[1ab][1Ce]+1a

where:

*X* = mass of adsorbed solute (adsorbate) (mg),
*M* = adsorbent weight (g),
*C_e_* = equilibrium concentration of adsorbate (mg/L),
*a* = maximum number of moles adsorbed per mass of adsorbent and
*b* = equilibrium constant of adsorbate in a solution after adsorption (L/mg).

Freundlich’s model (Equation (3)) is commonly used to describe wastewater treatments on activated carbon adsorption columns [25]. It is an empirical model that explains heterogeneous surface adsorption in which the concentration of the adsorbate in the adsorbent increases with increasing initial concentration of the solution [24]. This nonlinear model is more complex compared to other models; however, it generates much more reliable results [26].

(3) $$\frac{X}{M}=kC^{\frac{1}{n}}$$

This equation can be theoretically derived by assuming that the surface contains distinct types of adsorption centers. The adjustment parameters k and n are constant. They can be obtained from the linear fit of the expression in logarithmic form (Equation (4)).

(4) $$\ln\left[\frac{X}{M}\right]=\ln\left(k\right)+\frac{1}{n}\ln C$$

where

*X* = *C*_0_ − *C*,
*X/M* = retained adsorbate (mg),
*C*_0_ = initial concentration of adsorbate (mg/L),
*C* = final concentration (mg/L),
*M* = mass of adsorbent (g),
*k* = empirical constant (y intercept of the linear equation) and
*n* = constant (slope m of the linear equation).

Another model found in the literature is Temkin’s (Equation (5)). This model considers that, for high or low concentrations of adsorption between the adsorbent and the adsorbate, the heat of adsorption of all the molecules in the layer decreases linearly with coverage [27,28]. The model is used for systems whose adsorption enthalpy decreases linearly with θ, a factor that is not taken into account in the Langmuir isotherms. The linear representation θ versus ln (*C_e_*) (Equation (5)) allows obtaining *A* of the slope and *B* of the ordinate at the origin [25].

(5) $$\theta =B\ln A+B\ln C_{e}$$

where

*B* = (*RT*)/*b_T_*,
*T* = absolute temperature in Kelvin,
*R* = gas constant, 8314 J/molK,
*C_e_* = equilibrium concentration of adsorbate (mg/L),
*b_T_* = constant related to heat of adsorption and
*A* = balance constant (L/min) corresponding to the maximum compulsory energy.

By combining intensive Fenton oxidation (with which large molecules are converted into smaller ones and, therefore, easier to treat) with the adsorption process, over 99% COD and color removals are achieved [1]. On the other hand, it is necessary to optimize this last part of the process to observe: first, the interaction of the degraded leachate with Fenton in the adsorbent material and, second, if the nature of the adsorbent influences the efficiency of removal of organic material and inorganic. For these reasons, the aim of this study is to evaluate the efficiency of two different activated carbons, granular (GAC) and powdered (PAC), for use on in the adsorption stage included in the Fenton-adsorption process for landfill leachate.

The hypothesis of this work is that the PAC adsorption process preceded by Fenton can efficiently remove organic matter from the leachates, because the macromolecules of the crude leachate have been degraded to smaller molecules, thus preventing them from becoming clogged interstices of the PAC (due to its particle size, has a greater surface area than the GAC), allowing a better removal of organic matter and color.

## 2. Results

Table 1 presents the results of the tests with granular activated carbon (GAC) and powdered activated carbon (PAC), using as samples the raw leachate (RL) and the Fenton effluent (FE).

### 2.1. Adsorption Models with Activated Carbon

When a molecule is adsorbed, it can spread (move) on the surface, become fixed, undergo a chemical reaction (heterogeneous catalysis) or dissolve inside the solid (absorption). If a molecule has an affinity for activated carbon, it will be attracted to the adsorbent through different mechanisms (physisorption, chemisorption or both), until it reaches equilibrium. Adsorption isotherms are indicators that provide information on the adsorption capacity and the relationship of the adsorbent with the adsorbate [29,30]—in this case, how activated carbon works by adsorbing contaminants from the leachate.

**Figure 2:**
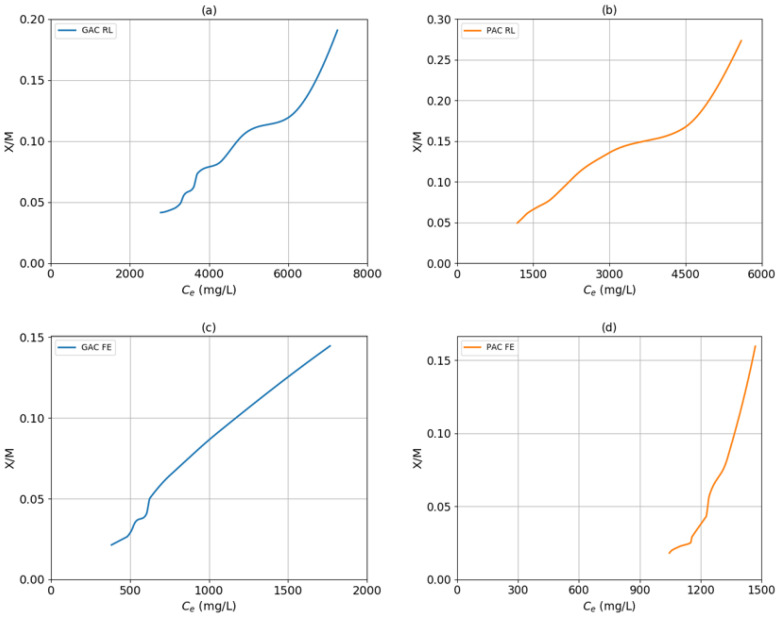
Experimental isotherms: granular activated carbon with raw leachate (GAC RL) (**a**), powdered activated carbon with raw leachate (PAC RL) (**b**), granular activated carbon with Fenton effluent (GAC FE) (**c**) and powdered activated carbon with Fenton effluent (PAC FE) (**d**).

Figure 2 shows the experimental isotherms of granular activated carbon with raw leachate (GAC RL), powdered activated carbon with raw leachate (PAC RL), granular activated carbon with Fenton effluent (GAC FE) and powdered activated carbon with Fenton effluent (PAC FE). The behavior of the curves for both samples and both carbons corresponds to a sigmoid isotherm (type II), since the curves are concave upwards, so multilayer adsorption is carried out [31,32] on the surface of activated carbon. With this information, it is possible to assume that the COD retained in the adsorbent occurs by physisorption, regardless of the nature and size of the activated carbon particle, regarding the leachates.

Adsorption isotherms describe how many contaminants interact with the adsorbent material. The easiest solutes to adhere to are the most molecularly complex compounds. Therefore, the adsorption process is widely used for the treatment of contaminated effluents [29,33]. If the polluting compounds are mixed in the effluent and move into it in an advection process, it is possible that the affinity of these substances with the adsorbent is high, as long as a steric effect does not occur in which the adsorption of the adsorbate caused by the presence of a functional group (that promotes a chemical reaction).

The data were analyzed according to the Langmuir, Freundlich and Temkin isotherm models, and the determination coefficients (R^2^) obtained are shown in Table 2. In all cases, an R^2^ with a high value is presented, so the models fit the data well. However, it is notable that the Freundlich and Temkin models fit better than the Langmuir model, showing that physical adsorption is taking place on the adsorbate (for both samples). Regarding the coefficients of determination, the PAC RL and GAC FE samples fit the Freundlich model better, while the GAC RL and PAC FE samples fit better with the Temkin model.

Freundlich’s model is valid for heterogeneous surfaces (such as those of activated carbon) and predicts an increase in the concentration of ionic species adsorbed on the surface of the solid when the concentration of certain species in the liquid phase increases [28]. It is the most commonly used model for residual effluents subjected to a type of adsorption, especially to remove organic compounds with activated carbon. This model describes nonideal reversible adsorption not restricted to monolayer formation (also applies to multilayer adsorption, with an uneven distribution of adsorption heat and affinity on the heterogeneous surface). The amount adsorbed is the sum of the adsorption at all the sites with the highest bond strength to be occupied first, until the adsorption energy decreases exponentially and the adsorption process is completed [34].

In the case of the study samples, the model indicates that adsorption is favorable in activated carbon, since they have an affinity and are easy to adsorb at the available sites of the adsorbent, forming layers (physisorption). This explains what happens in the adsorption of contaminants in the leachate samples (with both carbons). The value of n (shown in Table 2) indicates the adsorption intensity [6,24], PAC RL = 0.87 and GAC FE = 0.75 (n is a nondimensional number). The closer the value of n is to one, the adsorption will be linear; a value less than unity exhibits favorable adsorption, while when adsorption is greater, it is unfavorable [35].

It should be noted that the other samples (GAC RL and PAC FE) also present high coefficients of determination for the Freundlich model; however, the largest R^2^ was with the Temkin model. This model indicates that the adsorption heat (temperature) of all the molecules in the layer decreases linearly as it is covered; that is to say that, even when the isotherm test is controlled with the temperature variable, it will decrease as they saturate the carbon. Here, it is possible to appreciate, comparing both isotherms (Figure 2a,d), that the highest remaining C_e_ (measured as mg of COD/L) is for the GAC RL—that is, compared to PAC FE, the sample that supports more adsorption of pollutants (in a certain time). This may be due to the affinity of the leachate with the type of carbon used.

Under Table 2, the adsorption energy is 231.67 and 61,686 kJ/mol for GAC RL and PAC FE, respectively, indicating that in the first one more energy is released. Considering that, the lower the adsorption energy involved in the process, the greater the amount of adsorbed, it is possible to conclude that the contaminant removal from RL implies greater energy, since it is a solution with more molecules than FE, whose organic load has decreased in the process before to adsorption. Since the adsorbate particles are also different, comparing the other samples, it is possible to conclude that they act in the same way, depending on the sample (and does not depend on the activated carbon).

### 2.2. Organic Matter Removal Efficiency 

Using GAC is effective to treat mature leachates [36]. As observed in Table 1, using GAC the maximum COD removal from the RL (74.89%) is achieved with the highest dose; however, color removal is not adequate (0.45%). Using PAC with RL removes up to 89.29% COD (with the highest dose), and large color removals are achieved, greater than 94% from the 4-g dose, suggesting that PAC is best for removing contaminants from raw leachate. In general, activated carbon both granular and powder is used to treat leachates; however, the granular is mostly applied with better efficiencies without previous treatments [37].

In the FE sample treated with GAC, COD and color removals are greater than 90% and 97% (Table 1), respectively. However, when FE is treating with PAC, the COD removal efficiency reaches 90% with higher doses (8, 9 and 10 g), although the color removal is higher than the GAC treatment, since higher removals (99%) are obtained from the lowest dose (1 g).

With the data obtained (Table 2), the variance (95% confidence level) for the COD removal efficiency was analyzed, having, as independent variables, the sample, the type of carbon and the dose; this is in order to find similarities or differences in the studied effluents. This result is shown in Table 3, where it is possible to see that all the variables have a *p*-value <0.05, which means that they all affect the response variable.

In the same way, the analysis of variance was performed for color removal (Table 4), where the significant variables turned out to be the type of sample and the type of carbon, which means that they have a significant effect on the removal of color.

**Figure 3:**
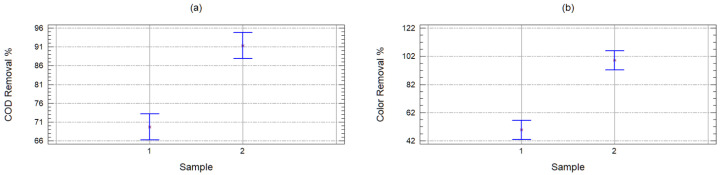
Mean charts of the chemical oxygen demand (COD) (**a)**) and color (**b**) removal efficiency, according to the type of sample (1 = RL and 2 = FE).

Figure 3 shows the mean charts, where is observed that the samples are indeed statistically different, and the one with the best removals is with the FE sample. With these results, it can be concluded that, for the leachate treatment, it is possible to obtain greater contaminant removals using the Fenton process prior to adsorption.

**Figure 4:**
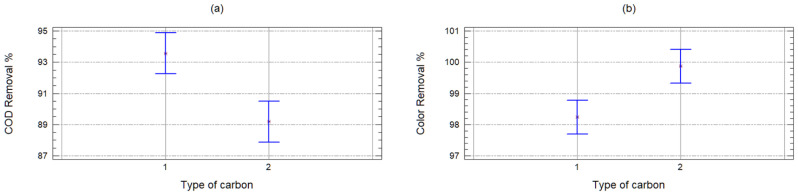
Mean charts of the COD **(a)** and color **(b)** removal efficiency in the FE, according to the type of carbon (1 = GAC and 2 = PAC).

When observing that the samples are different, it was decided to remove this variable from the analyses of variance. It was proceeded to continue analyzing only the results of the FE samples. According to the *p*-values (less than 0.05) obtained from the analysis of variance, both variables (carbon and quantity) turned out to be significant, and according to Figure 4, the carbon with which the best COD removals are obtained is with the GAC, while with the PAC, the color is removed more efficiently. This is for the characteristics of the carbons, since their pore size is different (macroporous GAC and mesoporous PAC) and have a different surface area, being greater in the mesoporous. Therefore, with this carbon, the larger molecules are better removed that have remained after intensive oxidation; however, it lets through those tiny ones that continue to confer the organic load to the effluent (humic substances).

**Figure 5:**
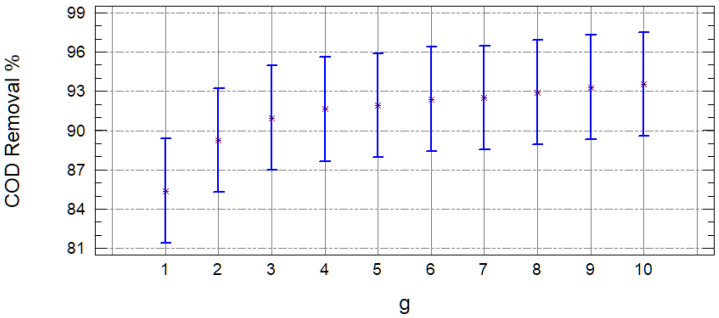
Mean charts of the COD removal efficiency in FE, according to the amount (g) of carbon used.

Figure 5 shows that, from the 2 g of carbon added, all the treatments are statistically the same; however, lower doses (1 g) offer lower COD removals. The adsorption power of GAC is for its high porosity and thermostability characteristics [36]. In general, activated carbon can remove recalcitrant compounds from leachates—both organic and inorganic—turbidity and color [38].

**Figure 6:**
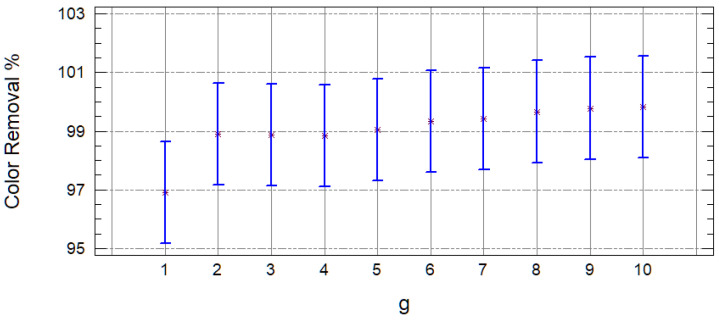
Mean chart of the color removal efficiency in FE, according to the amount (g) of carbon used.

In the analysis of variance of the color removal, with respect to the amount of carbon, a *p*-value equal to 0.999 is obtained, which indicates that all the amounts of carbon result in a statistically equal % removal of color. This can be observed in Figure 6. Kulikowska et al. 2016 [38] mention that large doses of PAC could be less efficient due to the high consumption of carbon related to a slight increase in efficiency.

### 2.3. Design of the Adsorption Column

With the results obtained, the granular was selected as the best activated carbon for the Fenton-adsorption process, since it offers greater COD removals. For the design of the adsorption column, the saturation test of a column packed with GAC was carried out using FE. 

**Figure 7:**
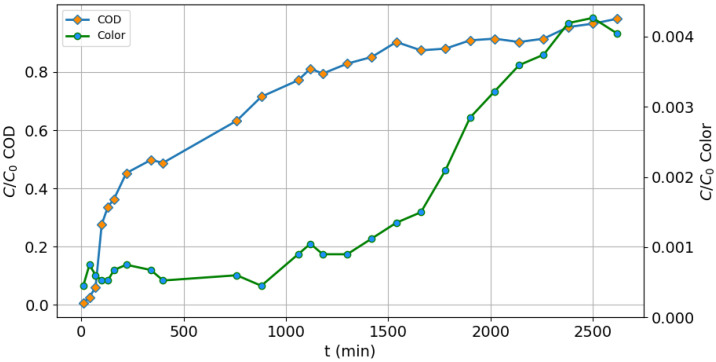
Saturation curve for the COD and color (GAC FE).

The saturation curve is shown in Figure 7, where it is observed that, during the first few minutes, activated carbon can remove the COD almost entirely, since the remainder (C/C_0_) approaches to zero. This value increases as the time in which the leachate continues to pass through the column until it is saturated. The saturation point was considered being the one where the COD concentration (C) was almost the same as the initial one (C_0_), which occurred at the 2620 minute, with a total treated volume of 445.4 L. This behavior is expected, since the molecules of the COD (although they have been oxidized with the Fenton process, they remain macromolecules) are retained in the interstices of the carbon, until they occupy all the available sites.

However, for the retention of the molecules that give color to leachate, the carbon was able to maintain a C/C_0_ < 0.005 until the end of the test, as observed in Figure 7. This is because, during Fenton oxidation, the dissolved solids in the leachate were degraded, so the size of these particles is much smaller than the original (of the crude leachate), and they are more easily adsorbed in the interstices of the carbon.

Table 5 shows the design parameters of the column, where it is indicated that 21.68 kg of COD can be removed from the leachate treated with Fenton for each kilogram of GAC, which makes its use viable in the treatment train. The removal of color and the COD are >99%; thus, it is concluded that the GAC is the best option for packing the adsorption column in the Fenton-adsorption process.

Table 6 presents a compilation of the treatments for leachates, in which advanced oxidation and/or adsorption processes are used with combined traditional or innovative mechanisms. As can be seen, the COD removal efficiencies obtained in this study are superior.

## 3. Materials and Methods 

### 3.1. Selection of Carbons

Two types of activated carbon were tested to determine the most efficient for removing organic matter (measured as COD and color) from leachates:

- Macroporous lignitic granular activated carbon (GAC). Gama L brand; raw material: lignite mineral (lignite); mesh number: 8 × 30 and surface area of 348.61 m^2^/g, relative density of 0.38 and cross-section of the adsorption area of 0.162 nm^2^.
- Powdered activated carbon (PAC) from mesoporous coconut shell. Micropol brand; raw material: coconut shell; mesh numbers: <50, <150 and <325 and holding capacity: between 0.2 and 1 kg of contaminants per kg of activated carbon.

Both carbons were subjected to the same process (see Section 3.3).

### 3.2. Leachate Samples

Leachate from the landfill of the City of Merida, Yucatan, Mexico was used in this study. Samples were taken to carry out the adsorption process in the raw leachate (RL) and in the effluent of the leachate treated with the Fenton process (FE). COD and color parameters were measured in the samples according to the standard method [49].

#### Fenton Process

It was carried out the treatment cited by San Pedro et al. 2015 [1] for the RL samples, which comprised adjusting the pH to 4 using concentrated H_2_SO_4_, carrying out the Fenton reaction with the ratios (Fe^2+^/H_2_O_2_) = 0.6(Fe_2_SO_4_·7H_2_O) and (COD/H_2_O_2_) = 9, H_2_O_2_ 30% *w*/*w* and contact time of one hour. Subsequently, the treated leachate was filtered with Whatman #40 filters (110-mm-diameter). The effluent from this treatment is the one used for the adsorption tests (FE).

### 3.3. Adsorption Test

To determine the removal efficiency of organic matter measured as the COD and color, the adsorption process was tested in triplicate with the two activated carbons as follows:

- In samples of 50 mL of leachate, amounts of 1 to 10 grams were added and stirred for one hour using stir plates.
- Subsequently, they were filtered with Whatman #40 filters to separate the carbon from the leachate, and the COD concentration and color were determined.

The data obtained was analyzed using the following statistical model:

*y_ij_* = μ + α*_i_* + β*_j_* + ε*_ij_* (6)

where

*y_ij_* = removal percentage (COD and color),
μ = great mean of the response variable,
α*_i_* = effect of GAC or PAC on the response variable,
β*_j_* = covariable effect of the dose of coal on the response variable and
ε*_ij_* = random error (due to variability in leachate composition and laboratory errors).

For all statistical analyzes, the STATGRAPHICS program, version 5.1 for Windows, factorial variance analysis and Fischer’s method of minimum significant difference (DMS) were used to contrast the means of each treatment.

### 3.4. Determination of the Adsorption Model 

The data obtained in 3.3 were used to determine the adsorption models (Langmuir, Freundlich and Temkin) by means of the analysis proposed in Table 7. Each test was carried out in triplicate, so the data in Table 1 are averages. The parameters presented in Table 7 are described below:

- Carbon mass (M). Depending on the type of carbon used (GAC or PAC), the doses range from 1000 mg to 10,000 mg, varying from thousand to thousand.
- Final COD (C_e_). For both samples (RL and FE), the COD was measured after treatment with carbon.
- Mass of the adsorbed solute (X). The ratio between the initial COD minus the final COD, divided by proportional volume.
- mg/mg ratio (X/M). Product of the mass of the adsorbed solute divided by the mass of the carbon used in the treatment.

### 3.5. Packed Column Adsorption 

To design the adsorption column in the Fenton-adsorption process, the evaluation of the adsorption behavior in continuous flow was carried out. A column was packed that operated under the conditions described in Table 8.

Samples of the effluent were taken from the column at different times to measure the COD concentration and color until saturation of the activated carbon was reached (this occurs once the COD concentration at the exit of the column is equal to the initial). With the data obtained in this test, the treatment rupture curve was elaborated, and the design parameters were established.

## 4. Conclusions

Granular activated carbon (macroporous) is more efficient in removing organic matter measured as the COD in the leachate treated with the Fenton process, while powdered activated carbon (mesoporous) removes its color more efficiently. The surface area of mesoporous carbon is greater than that of macroporous carbon, but the latter has a greater capacity of adsorbing larger molecules than mesoporous carbons. If all the molecules were small, mesoporous carbon would be more efficient. In raw leachate, there are molecules of diverse sizes, making the use of macroporous carbon more efficient. Once the Fenton process has been carried out, the macromolecules have partially oxidized, giving rise to smaller molecules that can be adsorbed by mesoporous or macroporous carbons.

Regarding the modeling of the isotherms, the samples of crude leachate treated with GAC and that of the Fenton effluent treated with PAC are adjusted to the Temkin model (R^2^ equal to 0.98 and 0.99, respectively). For the PAC-treated crude leachate and the GAC-treated Fenton effluent, the model that best fits the data is that of Freundlich (R^2^ equal to 0.99 and 0.97, respectively), which concludes that the adsorption of contaminants on the carbons is carried out by multilayers, through the physisorption of molecules related to the active sites of activated carbon. With the proposed GAC packed adsorption column, it can be removed 21.68 kg COD/kg carbon and color and COD removals greater than 99%. In conclusion, GAC has proven to be a highly efficient material for the treatment of leachate in the Fenton-adsorption process.

## Figures and Tables

**Table 1:** Average test results for granular activated carbon (GAC) and powdered activated carbon (PAC).

Sample	COD (mg/L)	S.D.	% Removal	Color (Platinum Unit Pt-Co)	S.D.	% Removal
M1C1d1	7247.5	110	34.49	13,150	778	4.02
M1C1d2	6147.5	343	44.41	12,950	212	5.37
M1C1d3	4827.5	265	56.35	11,300	566	17.27
M1C1d4	4330	198	60.85	11,200	566	18.00
M1C1d5	3710	396	66.44	10,200	707	25.57
M1C1d6	3610	304	67.35	10,850	495	20.57
M1C1d7	3355	7	69.67	11,550	1626	15.22
M1C1d8	3272.5	74	70.41	11,650	1909	14.43
M1C1d9	3087.5	95	72.09	13,850	5162	-2.33
M1C1d10	2777.5	53	74.89	13,450	5869	0.45
M1C2d1	5597.5	329	49.41	6400	283	53.15
M1C2d2	4450	870	59.82	4000	283	70.70
M1C2d3	2975	417	73.13	2435	658	82.33
M1C2d4	2352.5	124	78.74	745	92	94.57
M1C2d5	2047.5	95	81.50	1125	601	91.65
M1C2d6	1817.5	18	83.57	711.5	408	94.71
M1C2d7	1555	49	85.95	543.5	250	95.97
M1C2d8	1370	0	87.62	310.5	23	97.73
M1C2d9	1272.5	53	88.49	337.5	110	97.51
M1C2d10	1185	49	89.29	238	130	98.23
M2C1d1	1765.5	162	84.05	805	106	94.09
M2C1d2	1047	52	90.53	278	115	97.99
M2C1d3	758.5	40	93.14	286.5	13	97.91
M2C1d4	626.5	16	94.34	299.5	46	97.80
M2C1d5	604.5	8	94.54	251	1	98.16
M2C1d6	527.5	4	95.23	164.5	28	98.79
M2C1d7	506.5	2	95.42	147	115	98.90
M2C1d8	477	4	95.69	76	18	99.44
M2C1d9	425	42	96.16	51.5	25	99.62
M2C1d10	383.5	54	96.53	41.5	16	99.69
M2C2d1	1468.5	5	86.72	39	20	99.71
M2C2d2	1332	103	87.96	27.5	4	99.80
M2C2d3	1241.5	19	88.78	23.5	4	99.83
M2C2d4	1226	20	88.92	17.5	6	99.87
M2C2d5	1184.5	21	89.29	11	1	99.92
M2C2d6	1157	38	89.54	16.5	8	99.88
M2C2d7	1150	42	89.60	10	1	99.93
M2C2d8	1091.5	80	90.13	16	13	99.88
M2C2d9	1058.5	111	90.43	9	6	99.93
M2C2d10	1045.5	121	90.54	4	0	99.97

COD = chemical oxygen demand, S.D. = standard deviation, M1 = raw leachate (RL), M2 = Fenton effluent (FE), C1 = GAC and C2 = PAC. d1 = 1 g, d2 = 2 g, d3 = 3 g, d4 = 4 g, d5 = 5 g, d6 = 6 g, d7 = 7 g, d8 = 8 g, d9 = 9 g and d10 = 10 g.

**Table 2:** Fitted model constants and determination coefficients for adsorption isotherms. R^2^ = coefficients of determination.

Sample	Langmuir			Freundlich			Temkin		
	a (mg/g)	b (mg/L)	R^2^	K (mg/g)	n	R^2^	A	B	R^2^
GAC RL	0.1339	8.55 × 10^−5^	0.9574	15.603	0.6392	0.9727	9.7786	231.67	0.9834
PAC RL	0.6859	5.88 × 10^−5^	0.9920	11.076	0.8737	0.9934	10.9851	131.56	0.9526
GAC FE	0.1074	4.39 × 10^−4^	0.9575	11.74	0.7508	0.9672	10.9272	44.135	0.9523
PAC FE	0.0079	6.75 × 10^−4^	0.9537	49.532	0.1531	0.9769	9.8878	61.686	0.9969

a = maximum number of moles adsorbed per mass of adsorbent and; b = equilibrium constant of adsorbate in a solution after adsorption (L/mg); n = constant (slope m of the linear equation), indicate adsorption intensity; B = adsorption energy (RT/b_T_); A = balance constant (L/min) corresponding to the maximum compulsory energy.

**Table 3:** Results of analysis of variance for COD removal efficiency.

Source	Sum of Squares	Degrees of Freedom	Half-Square	F Coefficient	*p*-Value
Sample	4688.74	1	4688.74	61.36	0
Carbon	341.348	1	341.348	4.47	0.0436
Quantity	2196.44	9	244.049	3.19	0.0088
Waste	2139.5	28	76.4109		
Corrected total	9366.03	39			

**Table 4:** Results of analysis of variance for color removal efficiency.

Source	Sum of Squares	Degrees of Freedom	Half-Square	F Coefficient	*p*-Value
Sample	24,304.4	1	24,304.4	43.62	0
Carbon	14,988.9	1	14,988.9	26.9	0
Quantity	958.521	9	106.502	0.19	0.9933
Waste	15,599.8	28	557.136		
Corrected total	55,851.6	39			

**Table 5:** Design parameters for the adsorption column.

Parameter	Quantity
COD affluent (mg/L)	1750
COD effluent to 10 min (mg/L)	11
% removal of COD	99.37
Color affluent (U Pt-Co)	13,356
Color effluent (U Pt-Co)	6
% de removal de color	99.96
kg COD_REMOVED_/kg of carbon	21.68
Liters of leachate/kg of carbon	62.36

**Table 6:** Comparison of the COD and color removal efficiencies with different treatments applied to landfill leachate.

Treatment	COD Removal %	Color Removal %	Reference
Electro-persulfate oxidation process	45.7	97.3	[39]
Heterogeneous Fenton	88.6	-	[40]
Electro-Fenton	93	92	[41]
Photo-electro-Fenton process	97	100	[42]
Electrocoagulation	94	-	[43]
Coagulation/flocculation and Fenton combined treatment	62	-	[44]
Electrocoagulation and biofiltration	63	-	[45]
Adsorption with limestone and zeolite	55	76	[46]
Adsorption with wastepaper sludge and activated carbon	85.9	-	[47]
Micro-peat and activated carbon composite	87	74	[48]
Adsorption with granular activated carbon	89	92	[1]
Fenton-adsorption process with granular activated carbon	99.3	99.9	This study

**Table 7:** Analysis of data obtained in the adsorption test.

Test	Mass Carbon (mg) M	Final COD (mg/L) C_e_	Mass of Adsorbed Solute (mg) X	Ratio (mg/mg) X/M
1	1000	C_e1_	X_1_	X_1_/M_1_
2	2000	C_e2_	X_2_	X_2_/M_2_
3	3000	C_e3_	X_3_	X_3_/M_3_
4	4000	C_e4_	X_4_	X_4_/M_4_
5	5000	C_e5_	X_5_	X_5_/M_5_
6	6000	C_e6_	X_6_	X_6_/M_6_
7	7000	C_e7_	X_7_	X_7_/M_7_
8	8000	C_e8_	X_8_	X_8_/M_8_
9	9000	C_e9_	X_9_	X_9_/M_9_
10	10,000	C_e10_	X_10_	X_10_/M_10_

**Table 8:** Characteristics of the packed column.

Characteristic	Value
Adsorbent mass (GAC)	7144 g
Column height	60 cm
Column diameter	20 cm
Flow	170 mL/min
Initial COD	1750 mg/L
Initial color	13,356 U Pt-Co
Volume of empty spaces	7650 mL
